# An fMRI Study of Adult Brain Cortical Activation Following Intensive Learning

**DOI:** 10.3389/fpsyt.2020.00115

**Published:** 2020-03-03

**Authors:** Ferihan Ahmed-Popova, Stefan Sivkov, Mariyan Topolov, Asen Beshkov

**Affiliations:** ^1^ Department of Anatomy, Histology and Embryology, Faculty of Medicine, Medical University - Plovdiv, Plovdiv, Bulgaria; ^2^ Department of Pharmacology and Drug Toxicology, Faculty of Pharmacy, Medical University - Plovdiv, Plovdiv, Bulgaria; ^3^ Department of Psychiatry and Medical Psychology, Faculty of Medicine, Medical University - Plovdiv, Plovdiv, Bulgaria; ^4^ Research Institute, Medical University - Plovdiv, Plovdiv, Bulgaria

**Keywords:** functional MRI, memory paradigm, brain activation, intensive learning, cognition

## Abstract

Functional imaging techniques, fMRI in particular, has given the possibility to investigate non-invasively the cognitive processes in healthy populations and different disorders concerning neuro-psychiatry, thus unfolding the concepts guiding diagnosis and patient management. Different brain structures seem to support different types of cognitive functions in particular learning and memory thus the neurobiological explanation of the retrieval of information is associated with knowledge of brain plasticity, memory circuits, synaptic neurotransmission and the modulation of glial cells. Consistent with fMRI investigations of memory systems we tested the dependability of a memory paradigm using heterogeneous memory stimuli in order to find the neurobiological basis that correlates with memory task performance. Our study resulted with statistical significant differences in brain activations across the block design contrasts in both occipital and temporal regions in 29 mentally healthy students during a memory paradigm performance after intensive learning. As functional magnetic resonance imaging has become an important and reliable tool for investigation of brain anatomy and its function in health and disease, it becomes clear that further research of neurobiological basis of cognitive and memory domains can clarify different diagnostic prototypes and thus explain the human brain impairments in neuropsychological patients, since these are characterized by various cognitive dysfunctions.

## Introduction

Functional imaging techniques, fMRI in particular, has given the possibility to investigate non-invasively the cognitive processes not only in healthy populations, but in different disorders concerning neuro-psychiatry, thus unfolding the concepts guiding diagnosis and patient management (1, 2). In this sense, the original definition of translational neuroscience suggests that fundamental neurobiological knowledge can explain the brain functioning in healthy and pathological conditions (3).

Human brain seems to be a dynamic structure, which is continuously changing in response to experiences (4, 5). The application of different stimuli to the brain during experimental studies and the evaluation of functional imaging results support the increasing evidence of activity-dependent neuroplastic changes of the human brain across the life-span (6, 7). Studying the cognitive functions and memory systems in particular may uncover the specific processes associated with aging and brain development (8).

The present study aimed to investigate the cortical brain activations in response to a memory paradigm performance during a functional MRI scan after intensive learning in healthy individuals.

## Materials and Methods

### Subjects

We conducted an fMRI scanning of 29 mentally healthy, right handed students of Bulgarian origin (15 males and 14 females with a mean age of 20.0 ± 2.0). The handedness was determined using the Edinburgh handedness inventory (9). In order to examine the brain functioning after intensive learning, the experimental procedure was applied just after one month summer examination session with a duration of average 8 h intensive studying per day and mean exams final grade of 5.50.

Exclusion criteria included signs or a history of first degree relative with mental retardation, somatic disorders with neurological components; developmental, learning or psychotic disorders (schizophrenia, affective disorders, etc.) under DSM-IV (10); an identifiable neurological disorder (seizure disorder, head injury, multiple sclerosis etc.); a history of drug or alcohol abuse.

The study was carried out under the recommendations and protocol approval by the committee of “Research Complex for Translational Neuroscience” of Medical University of Plovdiv, Bulgaria. We obtained a written informed consent from all participants in accordance with ethical principles of the Declaration of Helsinki.

### Experimental Procedure

The experimental procedure included a scan with a 3Т MRI system—GE Discovery 750w with a protocol of structural scan—Sag 3D T1 F-BRAVO FSPGR, slice thickness 1 mm, matrix 256 × 256, flip angle 10° and standard block design functional scan—2D EPI, slice thickness 3 mm, matrix 64 × 64, TR (repetition time)—3,000 msec, TE (echo time)—30, flip angle 90°.

**Figure 1:**
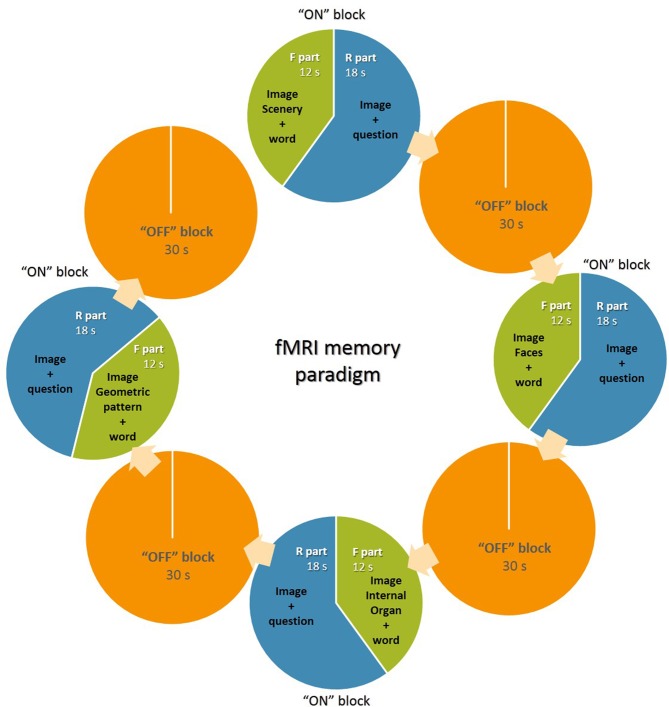
Graphical scheme of the experimental design of the fMRI memory paradigm.

Using a standard block design with total duration of 4 min, we defined four consecutive sets of “on” and “off” blocks. Participants were first presented with four consecutive pictures of the same theme—landscapes, portraits, anatomical images of internal organs and geometric figures denoted by specific mismatching nouns (seasons, personal names, internal organs, figures) (**fixation—F part**) with a duration of 3 s, followed by three of the pictures with questions (**recall—R part**) for memory evaluation (What was the word under this picture? Four possible answers.), for each of which the response time was 6 s. Subjects responded by pressing one of four possible buttons (two in each hand). The total duration of the active “on” blocks was 30 s. A rest—”off” block of 30 s followed each of the “on” blocks (**Figure 1**). The subjects were instructed to read carefully the questions and answer with a button press during the active conditions and to look at the central fixation cross without thinking of anything in particular for the resting conditions.

### fMRI Data Analysis

Functional MRI data analysis was done with SPM 12 (Statistical Parametric Mapping) software running on MATLAB R2015 for Windows. The preprocessing included five steps as follows: 1) realignment of the functional data for correction for head motion, 2) co-registration between the high-resolution anatomical image and the functional scans, 3) intra-individual estimation of spatial registration parameters based on the anatomical image, 4) transformation of the co-registered functional data to standardized MNI (Montreal Neurological Institute) space, followed by 5) spatial smoothing with a 6mm full-width-at-half-maximum Gaussian kernel.

We specified the first level analysis model, estimated and defined the parameters and t-contrasts for both active conditions vs. the passive condition (F > off, R > off), the first active condition vs. the second (F > R) and the opposite (R > F). The resulting contrast maps from each contrast and for each subject were then used in a second level random effects analysis for within group and between groups effects (males vs. females). Statistical significant level was set at P <0.05 Family Wise Error (FWE) corrected.

## Results

### fMRI Results

Our study resulted with statistical significant differences in brain activations across the block design contrasts in both occipital and temporal regions in males and females for two of the contrast maps—F > R and R > F.

**Figure 2:**
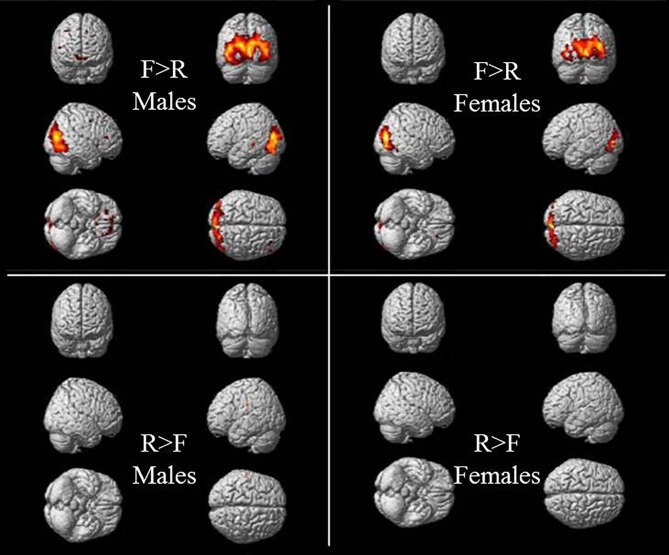
Significant residual activations of the F > R and R > F t-contrasts in male and female subjects after intensive learning.

Within both genders, the F > R contrast resulted in statistical significance of brain activations. We found significant clusters with peak activations in males in the region of left superior occipital gyrus (MNI coordinates −12, −88, 16) expanding to the right caudate nucleus (20, 16, 12) and left middle temporal gyrus (−54, −32, −2) (**Table 1**, **Figure 2**).

**Table 1:** Males peak-level in a one-sample t-test of the F > R and R > F contrast maps after intensive learning.

Anatomical localization peak activation	Cluster size (number of voxels)	pFWE-corr	MNI coordinates		
			x	y	z
**F–R**					
**Left superior occipital gyrus**	**12,583**	0.000	−12	−88	16
**Right caudate nucleus**	**2,348**	0.000	20	16	12
**Left middle temporal gyrus**	**51**	0.006	−54	−32	−2
**R–F**					
**Left postcentral gyrus and** **Left supramarginal gyrus**	**24**	0.022	−58	−20	30

The clusters of female participants located in the right middle occipital gyrus (34, −80, 12), right putamen (18, 12, −2) and left caudate nucleus (−16, 14, 0) showed significant residual activations as a result of the F > R contrast (**Table 2**, **Figure 2**).

**Table 2:** Females peak-level in a one-sample t-test of the F > R and R > F contrast maps after intensive learning.

Anatomical localization peak activation	Cluster size (number of voxels)	pFWE-corr	MNI coordinates		
			x	Y	z
**F–R**					
**Right middle occipital gyrus**	**6,041**	0.000	34	−80	12
**Right putamen**	**30**	0.008	18	12	−2
**Left caudate nucleus**	**22**	0.011	−16	14	0
**R–F**					
**Right precuneus**	**5**	0.031	14	−58	30
**Right supramarginal gyrus**	**5**	0.039	58	−24	28

Significant BOLD-signal differences were found in the R > F contrast with clusters in left postcentral gyrus and left supramarginal gyrus (−58, −20, 30) in males (**Table 1**, **Figure 2**). Females showed peak activation of the R > F contrast in the right precuneus (14, −58, 30) and right supramarginal gyrus (58, −24, 28) (**Table 2**, **Figure 2**).

Correction for multiple comparisons failed in finding significant residual activations for the F > R and R > F contrasts when applied in between-group analysis of males vs. females.

## Discussion

Consistent with similar studies of functional magnetic resonance imaging performance of memory tasks we determined the reliability of a memory paradigm in an experimental investigation of adult brain cortical areas that correlate with memory encoding after intensive learning in healthy individuals from both genders. Despite we did not find significant distinctions in the brain activations between males and females for the F > R and R > F contrasts, the within group results showed statistically significant differences for both the contrast maps.

The occipital residual brain activations of the F > R contrast in both genders is explicable due to the presence of visual stimuli during the fixation part. The same contrast map showed significant differences in the peak activation with clusters in right caudate nucleus and left middle temporal gyrus in males and right putamen and left caudate nucleus in females. Consistent with other findings (11, 12), our results indicate that memory encoding during spontaneous memory tasks including visual stimuli involve both sensory and non-sensory brain areas. Thus, occipital–temporal lobe regions are considered to participate in associative recognition and item recognition (13), and their dynamic connectivity with other cortical areas, such as basal ganglia, is essential in the memory process (14). Several studies highlighted the relevance of the striatal gray matter volume for memory and working memory operations (15, 16). A research report of Bauer et al., 2015 revealed “the relevance of caudate for associative memory decline in the aging brain” suggesting that the “right striatum (putamen and caudate) was correlated with recognition accuracy”, whereas the “bilateral caudate was positively associated with associative learning accuracy” (17).

Furthermore, the significant peak activations of the R > F contrast resulted in clusters at the supramarginal gyrus in both males and females, although in women, due to the small number of clusters, the visualization of brain activity is almost impossible (**Figure 2**). The association brain cortex at the parietal areas of right and left hemispheres and their inferior regions in particular, including the supramarginal gyrus, mediate somatosensory integration, representation and organization of movements, motor attention and identification of motor gestures, as well as in visual word recognition (18). It is considered that left supramarginal gyrus plays a key role of the short-term memory network, preserving an abstract representation of information from serial orders, regardless of content information (19–21). As functional magnetic resonance imaging has become an important and reliable tool for investigation of brain anatomy and its function in health and disease, it becomes clear that further research of neurobiological basis of cognitive and memory domains can clarify different diagnostic prototypes and thus explain the human brain impairments in neuropsychological patients, since these are characterized by various cognitive dysfunctions (22, 23).

However, our study has several limitations. Greater sample size is probably necessary to find statistical significant differences between the genders when comparing the BOLD-activations. We found similar imaging results in the active brain regions in one of our previous studies applying the same memory paradigm and same volunteers during a period of time when the students were not subjected to intensive learning (24). Therefore, further research and statistical analysis is indispensable for evaluation of the specific differences in cortical activation areas between both conditions in order to contribute to the disclosure of the way brain works in health and disease.

## Conclusion

The results of the present study support the recent investigations on brain plasticity and memory systems as an experimental approach in the multidisciplinary essence of translational neuroscience. Despite the limitations that should be considered in future research, the present study corroborates the findings that human brain is a dynamic structure, which reacts with complex interactions in both cortical and subcortical regions with regard to memory stimuli and tiny areas with subtle activations as supramarginal gyrus seem to play an important role in short-term memory circuits. Considering the results obtained after intensive learning and previous similar studies of brain activations at rest, we suppose that a further in-depth comparative analysis would enrich the data on how the human brain responds to stress, which in turn would be important in evaluating brain plasticity and function in disease.

## Data Availability Statement

The datasets generated for this study are available on request to the corresponding author.

## Ethics Statement

The studies involving human participants were reviewed and approved by Committee on Scientific Ethics, “Research Complex for Translational Neuroscience”, Medical University of Plovdiv, Bulgaria. The patients/participants provided their written informed consent to participate in this study.

## Author Contributions

FA-P and SS designed and performed the experimental study. FA-P and SS processed the results and wrote the manuscript. MT and AB managed the literature searches. All authors have approved the final manuscript after revision.

## Funding

This work was made possible as a result from a project of the Medical University of Plovdiv, for “Research Complex for Translational Neuroscience”, established with a grant from Operative Program “Competitiveness of Bulgarian Economy”.

## Conflict of Interest

The authors declare that the research was conducted in the absence of any commercial or financial relationships that could be construed as a potential conflict of interest.
